# Does health insurance impact health service utilization among older adults in urban China? A nationwide cross-sectional study

**DOI:** 10.1186/s12913-020-05489-8

**Published:** 2020-07-09

**Authors:** Wenhui Mao, Yaoguang Zhang, Ling Xu, Zhiwen Miao, Di Dong, Shenglan Tang

**Affiliations:** 1 Duke Global Health Institute, Duke University, Durham, NC 27705 USA; 2 Centre for Health Statistics and Information, National Health Commission, Beijing, China; 3 School of Public Policy and Administration, Xi’an Jiaotong University, Xian, China; 4 Health Human Resources Development Centre, National Health Commission, Beijing, China; 5 Global Health Research Center, Duke Kunshan University, Kunshan, Jiangsu China

## Abstract

**Background:**

China’s rapidly aging population has led to many challenges related to the health care delivery and financing. Since 2007, the Urban Residents Basic Medical Insurance (URBMI) program has provided financial protection for older adults living in urban areas not already covered by other health insurance schemes. We conducted a national level assessment on this population’s health needs and health service utilization.

**Methods:**

Records for 9646 individuals over the age of 60 were extracted for analysis from two National Health Service Surveys conducted in 2008 and 2013. Multiple regression models were used to examine associations between socioeconomic factors, health needs and health service utilization while controlling for demographic characteristics and survey year.

**Results:**

Self-reported illness, especially non-communicable diseases (NCDs) increased significantly between 2008 and 2013 regardless of insurance enrollment, age group or income level. In 2013, over 75% of individuals reported at least one NCD. Outpatient services decreased for the uninsured but increased for those with insurance. Middle- and high-income groups with insurance experienced a higher increase in outpatient visits and hospital admissions than the low-income group. Forgone hospital admissions (defined as an admission indicated by a doctor but which was declined or not followed through by the patient) decreased. However, over 20% of individuals had to forgo necessary hospital admissions, and 40% of these cases were due to financial barriers. Outpatient visits and hospital admissions increased between 2008 and 2013, and insured individuals utilized more services than those without insurance.

**Conclusion:**

After the implementation of URBMI**,** health service utilization increased and forgone hospital admissions decreased, indicating the program helped to improve access to health services. However, there was still a marked difference in utilization among different income groups, with the high-income group experiencing the greatest increase. This factor calls for further attention to be given to issues related to equity. Prevalence of self-reported NCDs greatly increased among the study population between 2008 and 2013, suggesting that health insurance programs need to ensure they cover sufficient support for the treatment and prevention of NCDs.

## Background

Financial protection for healthcare is vital to achieving universal health coverage (UHC) globally [1]. China has made significant strides in establishing several health insurance schemes to improve financial protection for its population. The Urban Employees’ Basic Medical Insurance (UEBMI) (established in 1999) and the New Cooperative Medical Schemes (NCMS) (established in 2003) have successfully provided insurance coverage for employees (including retirees) in urban regions and all residents in rural areas, respectively. However, not all individuals fall into these categories, and the Urban Residents’ Basic Medical Insurance (URBMI) was established to close “the last mile” gap in UHC in China [2]. First piloted in 79 cities in 2007, URBMI was fully scaled-up across the country in 2009.

Adapting similar policies to those used by NCMS, URBMI targeted the elderly, unemployed and children (including students) not already covered by the other health insurance [3]. According to national records, URBMI covered 43 million people in 2007 and increased coverage to 271 million by 2012. As a result, over 95% of Chinese population have been covered by either UEBMI, URBMI or NCMS since 2010 [3]. URBMI is financed through individual premiums and government subsidies. In 2007, the minimal government subsidy was 40 CNY (6 US$) per person [4] which increased to 240 CNY (34 US$) per person in 2012 [5]. Insurance premiums vary by city and in general eastern cities provide higher premiums than western cities, which has the similar distribution to the regional economic development [6]. For example, in 2012 the government subsidy for URBMI in western cities followed the national threshold (240 CNY/ 34 US$) with 60 CNY (10 US$) from beneficiary, while the subsidy in an eastern city was 800 CNY (113 US$) and required the beneficiary to contribute 400 CNY (57 US$) [7]. The URBMI benefits package was established using the National Health Insurance Reimbursable List (NHIRL), and only medicines, diagnostic and treatment procedures and services listed in the NHIRL were eligible for reimbursement [8]. URBMI initially covered inpatient services and limited outpatient services covered to catastrophic illness (such as cancer). However, since 2011 coverage for outpatient services has been expanded [4, 9] and the reimbursement rate for inpatient services increased from 49% in 2008 to 54% in 2013 [10]. For more details on insurance policies we refer the reader to the People’s Republic of China health system review (Chapter 3 Health Financing) by the World Health Organization Regional Office for the Western Pacific. (Available at: http://iris.wpro.who.int/handle/10665.1/11408).

While URBMI provides additional coverage for China’s vulnerable population, its unique features create several challenges. First, one of the target populations of URBMI are older adults, a population with higher medical needs than the overall population [11, 12]. China has a rapidly aging population and individuals aged 60 years or over will make up 19.5% of the population by 2025 [13], resulting in increasing demands on health services. Second, different benefits packages between insurance schemes cause issues related to equity. UEBMI usually provides higher reimbursements in urban areas compared to URBMI but the beneficiaries of both programs utilize the same health care systems, leading to higher out-of-pocket payment for URBMI beneficiaries [14, 15]. Third, the recent integration of URBMI and NCMS was aimed to reduce urban-rural disparities yet its impact on health services utilization remains under-reported [16].

Limited evidence about the impact of URBMI makes addressing these challenges difficult. Chen, G. et al. found that URBMI had significantly increased the use of both inpatient and outpatient treatment but its impact on reducing forgone hospitalization was insignificant [17]. While Li, X. and colleagues found that URBMI’s impact on service utilization was not sufficient [15]. Evidence on the impact of URBMI on different income groups is also contradicted [4, 11, 18, 19]. Few studies have compared the difference between URBMI enrollees and uninsured members of the population in terms of health needs and service utilization.

This paper aims to assess the changes in health needs and health services utilization among older adults living in urban areas across China. Additionally, we explore the impact of URBMI and other factors on service utilization, with special attention given to different socio-economic and demographic groups.

## Methods

### Data

This study extracted data from two National Health Service Surveys (NHSS) conducted in 2008 and 2013. NHSS is a nationally representative cross-sectional household survey conducted every 5 years by the Center for Health Statistics and Information of National Health Commission (formerly known as the China National Health and Family Planning Commission). Using a multi-stage randomized sampling method, trained interviewers conducted face-to-face interviews with all available adult members of selected households using structured questionnaires. The questionnaires collected information on demographic and socio-economic status, health insurance enrollment, health needs, health service access and utilization, medical expenditures, and household expenditures. Detailed methodology on these surveys are available elsewhere [20].

Study participants included individuals aged 60 years and over who were eligible for URBMI regardless if they were enrolled or not. As URBMI was just piloted in 2007, we only extracted data from the 2008 survey in the URBMI pilot cities. However, we extracted data from all urban cities from the 2013 survey. A total of 9646 eligible records were extracted, 2103 from 2008 and 7543 from 2013. The number increased between the two surveys due to the scale up of URBMI between 2008 and 2013.

### Indicators

Health needs were determined using participants reported perceptions about their own health, and their belief that seeking health care was necessary whether or not it actually was. Major indicators used to measure health needs included self-reported illness or injury in the past two weeks, and self-reported diagnosis of non-communicable diseases (NCDs). Indicators for health services utilization included any outpatient visits in the past two weeks, hospital admissions within the past year, proportion of forgone necessary hospital admissions, and whether or not forgone admissions were due to financial difficulties. A forgone necessary hospital admission is defined as a hospital admission indicated by a doctor but which was declined or not followed through by the patient.

Individual level data were collected on age, gender, marital status, highest level of education and health status. Health status was measured using the EuroQol survey instrument (EQ-5D), comprising of five health modalities including mobility, self-care, daily activities, pain/discomfort, and anxiety/depression. EQ-5D index, ranged from 0 to 1 and higher score indicated better overall health, was then calculated and reported in this study [21]. Household size, annual household income and source of income were collected at the household level. To determine individual level of income, we divided the household income by household size. Income groups were divided based on the annual income per person. Income level was adjusted for Consumer Price Index (CPI) using 2008 as reference level and the adjustment ratio for 2013 was 117.5%.

### Analysis

To observe the differences between individuals with or without insurance, we stratified all analysis by insurance status. Indicators were analyzed by age groups (60–69 years, 70–79 years, 80 years or over) and income levels (low, middle, and high). Statistical significance was examined using Chi square tests and t tests.

Five logistic regression models were used to estimate associations between socioeconomic factors (age and income), health needs and health services utilization (EQ5D, difficulty in self-care) while controlling for demographic characteristics (sex, household size, marriage status, and education,) and survey year. Models 1 and 2 aimed to explore the impact factors on services use for insured population. Therefore, both models used records from insured individuals and the independent variables for models 1 and 2 represented outpatient (whether the individual had an outpatient visit in the past two weeks, 0 for “no” and 1 for “yes”) and inpatient services (whether the individual had received inpatient services in the past year,0 for “no” and 1 for “yes”), relatively. Model 3-5 used records from the entire study population to explore the impact of insurance on services use by introducing a dummy variable of insurance status (0 for “uninsured”, and 1 for “URBMI”). Similarly, model 3 used outpatient service and model 4 used inpatient as independent variables. Model 5 was built on Model 4 but added additional variable of self-reported NCDs. All of the five models shared the same set of dependent variables: age group (60–69 as base) and income groups (lowest as base), income source (other as base level, such as from children, friends), difficulty in self- care in the past month (no difficulty as base level), EQ-5D, sex (woman as base), household size (1–2 persons as base), marriage status (single or widowed as base level), education (none and primary as base level), and survey year (2008 as base).

## Results

### Demographic characteristics

Unsurprisingly, there were differences in demographic characters between the insured and uninsured groups. The proportion of men (*p* < 0.05 for URBMI), those aged 60 to 69 (*p* < 0.05 for URBMI), who were married (*p* < 0.05 for both uninsured and URBMI), with secondary level or higher education (*p* < 0.05 for both uninsured and URBMI), and relying on their own income rather than support from children (*p* < 0.05 for both uninsured and URBMI) increased from 2008 to 2013 (Table 1). The health status measured by EQ5D and the annual income per capita (*p* < 0.05 for both uninsured and URBMI) also increased from 2008 to 2013.

**Table 1:** Demographic characters

	2008		2013	
	Uninsured	URBMI	Uninsured	URBMI
**N (individual)**	1288	815	724	6819
**Household size**	3.1	3.0	2.9	3.1
**Sex #**				
Men	448 (34.8%)	255 (31.3%)	279 (38.5%)	2429 (35.6%)
Women	840 (65.2%)	560 (68.7%)	445 (61.5%)	4390 (64.4%)
**Age group##**	**	**	**	**
60–69	538 (41.8%)	356 (43.7%)	321 (44.3%)	3943 (57.8%)
70–79	457 (35.5%)	322 (39.5%)	233 (32.2%)	2015 (29.5%)
80+	293 (22.7%)	137 (16.8%)	170 (23.5%)	861 (12.6%)
**Marital status††##**			**	**
Single or widowed	562 (43.6%)	319 (39.1%)	233 (32.2%)	1827 (26.8%)
Married	721 (56.0%)	490 (60.1%)	488 (67.4%)	4983 (73.1%)
Others	5 (0.4%)	6 (0.7%)	3 (0.4%)	9 (0.1%)
**Highest education††##**	**	**	**	**
None and primary	1009 (78.4%)	600 (73.7%)	430 (59.4%)	4539 (66.6%)
Secondary	267 (20.7%)	194 (23.8%)	231 (31.9%)	2147 (31.5%)
College and above	11 (0.9%)	20 (2.5%)	63 (8.7%)	133 (2.0%)
**Per capita annual income (CNY, mean) ††##**	6296**	8352**	14,151**	12,260**
**Per capita annual income (CNY, median)**	5000	7200	11,064	10,213
**EQ-5D**	0.867	0.874	0.881	0.889
**Difficulty in self-care in the past month**	254 (19.7%)	150 (18.4%)	109 (15.1%)	864 (12.7%)
**Income resource††##**	**	**	**	**
Own	608 (47.2%)	460 (56.4%)	448 (61.9%)	4653 (68.2%)
Others	680 (52.8%)	355 (43.6%)	276 (38.1%)	2166 (31.8%)

* Significant difference between uninsured and URBMI; * for *P* < 0.05, ** for *P* < 0.01

†Significant difference for uninsured between 2008 and 2013, † for *P* < 0.05, †† for *P* < 0.01

#Significant difference for URBMI between 2008 and 2013, # for *P* < 0.05, ## for *P* < 0.01

Women comprised over 60% of the entire study population, and the number of men without insurance was higher than the number of men enrolled in URBMI, although the difference was not significant (*P* > 0.05). The insured group included a significantly higher proportion of those aged 60 to 69 (*p* < 0.05) and higher proportion of married individuals (*p* > 0.05) compared to the uninsured group. The insured group had a higher annual income per capita than the uninsured group in 2008 (*p* < 0.05) but the opposite trend (*p* < 0.05) was found in 2013. The insured group were more likely to have their own income rather than rely on support from children or others (*p* < 0.05) (Table 1).

### Self-reported health needs

Self-reported illness, especially NCDs, increased significantly between 2008 and 2013, regardless of insurance enrollment, age group or income level (*p* < 0.05 for all indicators among both insured and uninsured). The percent of individuals who reported an illness or injury within the past 14 days increased from about 40% in 2008 to over 64% in 2013. Over 75% of individuals reported having at least one NCD in 2013 (Table 2).

**Table 2:** Self-reported health needs

	Self-reported illness or injury in past 2 weeks					Prevalence of self-reported NCDs			
	2008		2013			2008		2013	
	Uninsured	URBMI	Uninsured	URBMI		Uninsured	URBMI	Uninsured	URBMI
**Total ††##**	509 (39.5%)	355 (43.6%)	463 (64.0%)	4553 (66.8%)	**Total ††##**	767 (59.5%)*	553 (67.9%)*	546 (75.4%)	5306 (77.8%)
**Sex ††##**					**Sex ††##**				
Men	140 (31.3%)	90 (35.3%)	196 (70.3%)*	1378 (56.5%)*	Men	237 (52.9%)	145 (56.9%)	226 (81.0%)*	1601 (65.9%)*
Women	369 (43.9%)	265 (47.3%)	267 (60.0%)*	3180 (72.4%)*	Women	530 (63.1%)*	408 (72.9%)*	320 (71.9%)**	3705 (84.4%)**
**Age group ††##**					**Age group ††##**				
60–69	181 (33.6%)	126 (35.4%)	160 (49.8%)	2413 (61.2%)*	60–69	297 (55.2%)	194 (54.5%)	205 (63.9%)	2821 (71.5%)
70–79	192 (42.0%)	155 (48.1%)	178 (76.4%)	1529 (75.9%)	70–79	300 (65.6%)*	256 (79.5%)*	208 (89.3%)	1795 (89.1%)
80+	136 (46.4%)	74 (54.0%)	125 (73.5%)	611 (71.0%)	80+	170 (58.0%)*	103 (75.2%)*	133 (78.2%)	690 (80.1%)
**Income group (per person) ††##**					**Income group (per person)††##**				
Low	197 (36.3%)	69 (39.4%)	147 (50.2%)	1842 (65.2%)**	Low	304 (56.0%)*	127 (72.6%)*	185 (63.1%)*	2132 (75.4%)*
Middle	161 (40.3%)	99 (36.7%)	73 (46.2%)	1508 (68.9%)**	Middle	250 (62.5%)	151 (55.9%)	93 (58.9%)**	1397 (80.1%)**
High	151 (44.0%)	187 (50.5%)	243 (89.0%)	1508 (67.1%)**	High	213 (62.1%)	275 (74.3%)	268 (98.2%)**	1777 (78.9%)**

* Significant difference between uninsured and URBMI; * for *P* < 0.05, ** for *P* < 0.01

†Significant difference for uninsured between 2008 and 2013, † for *P* < 0.05, †† for *P* < 0.01

#Significant difference for URBMI between 2008 and 2013, # for *P* < 0.05, ## for *P* < 0.01

Insured groups, women, and those in the high-income group had higher self-reported illness or injuries in the past two weeks, including a higher prevalence of self-reported NCDs in both years. In 2008, about 59.5% of the study population without insurance reported having an NCD while over 67% of individuals with insurance reported having an NCD (*p* < 0.05). With the scale up of URBMI by 2013, self-reported NCD prevalence was similar for both the insured and uninsured groups (*p* > 0.05) (Table 2).

### Health services utilization

There was a noticeable gap between individuals who reported needing health services and actually utilizing them (Tables 2 and 3). In addition, outpatient services decreased among the uninsured (*p* > 0.05) but increased for those with insurance between 2008 and 2013 (*p* > 0.05). Both insured and uninsured groups had an increase in hospital admissions during the same period although the change was not significant (*p* > 0.05 for both URBMI and uninsured) (Table 3).

**Table 3:** Health services utilization

	Outpatient visits in the past two weeks					Hospital admission within the past year			
	2008		2013			2008		2013	
	Uninsured	URBMI	Uninsured	URBMI		Uninsured	URBMI	Uninsured	URBMI
**Total**	388 (30.1%)	193 (23.7%)	181 (25.0%)	1842 (27.0%)	**Total**	127 (9.9%)	108 (13.3%)	85 (11.7%)*	1129 (16.6%)*
**Sex**					**Sex**				
Men	127 (28.3%)	50 (19.6%)	89 (31.9%)	637 (26.2%)	Men††	30 (6.7%)**	39 (15.3%)**	43 (15.4%)	346 (14.2%)
Women	261 (31.1%)	143 (25.5%)	92 (20.7%)	1025 (27.4%)	Women#	97 (11.5%)	69 (12.3%)	42 (9.4%)**	783 (17.8%)**
**Age group**					**Age group**				
60–69	143 (26.6%)	86 (24.2%)	80 (24.9%)	966 (24.5%)	60–69	40 (7.4%)	37 (10.4%)	34 (10.6%)	524 (13.3%)
70–79	139 (30.4%)	80 (24.8%)	63 (27.0%)	646 (32.1%)	70–79	49 (10.7%)	50 (15.5%)	31 (13.3%)*	446 (22.1%)*
80+	106 (36.2%)	27 (19.7%)	38 (22.4%)	230 (26.7%)	80+	38 (13.0%)	21 (15.3%)	20 (11.8%)	159 (18.5%)
**Income group (per person)**					**Income group (per person)**				
Low	145 (26.7%)	46 (26.3%)	66 (22.5%)	627 (22.2%)	Low	46 (8.5%)	27 (15.4%)	26 (8.9%)*	447 (15.8%)*
Middle	126 (31.5%)	62 (23.0%)	25 (15.8%)*	516 (29.5%)*	Middle	37 (9.3%)	30 (11.1%)	7 (4.4%)**	304 (17.4%)**
High	117 (34.1%)	85 (23.0%)	90 (33.0%)	699 (31.1%)	High	43 (12.5%)	51 (13.8%)	52 (19.0%)	378 (16.8%)

* Significant difference between uninsured and URBMI; * for *P* < 0.05, ** for *P* < 0.01

†Significant difference for uninsured between 2008 and 2013, † for *P* < 0.05, †† for *P* < 0.01

#Significant difference for URBMI between 2008 and 2013, # for *P* < 0.05, ## for *P* < 0.01

The insured group had a higher number of hospital admissions than the uninsured group in both 2008 (*p* > 0.05) and 2013 (*p* < 0.05). The insured group had more outpatient visits than the uninsured group in 2013 (*p* > 0.05) but the same was not true in 2008 (*p* > 0.05). Women tended to utilize more outpatient services than men, but no obvious pattern was observed between genders for hospital admission. Groups aged 60–69 and 70–79 had higher outpatient service utilization than those aged 80 and over, but those aged 70–79 and 80 and over had higher rates of hospital admission. In 2008, middle- and high- income groups with insurance experienced a higher increase in outpatient visits and hospital admissions than the low-income group. No such trend was observed among different income groups without insurance (Table 3).

The number of forgone necessary admissions and the number of those caused by financial difficulties decreased greatly between 2008 and 2013. However, in 2013, over 20% of individuals still had to forgo necessary admissions, and 40% of these were caused by financial barriers (Table 4).

**Table 4:** Forgone necessary hospital admission

	% of forgone necessary hospital admission				% of forgone necessary hospital admissions due to financial difficulties			
	2008		2013		2008		2013	
	Uninsured	URBMI	Uninsured	URBMI	Uninsured	URBMI	Uninsured	URBMI
**Total**	72 (36.2%)	45 (29.4%)	31 (26.7%)	243 (17.7%)	51 (86.4%)	35 (85.4%)	12 (52.2%)	85 (43.8%)
**Sex**								
Men	23 (43.4%)	17 (30.4%)	20 (18.9%)	79 (18.6%)	16 (84.2%)	12 (92.3%)	4 (50.0%)	22 (33.8%)
Women	49 (33.6%)	28 (28.9%)	21 (33.3%)	164 (17.3%)	35 (87.5%)	23 (82.1%)	8 (53.3%)	63 (48.8%)
**Age group**								
60–69	27 (40.3%)	15 (28.8%)	15 (30.6%)	128 (19.6%)	22 (84.6%)	11 (78.6%)	7 (63.6%)	48 (49.5%)
70–79	34 (41.0%)	21 (29.6%)	11 (26.2%)	88 (16.5%)	20 (87.0%)	16 (84.2%)	3 (42.9%)	29 (38.2%)
80+	11 (22.4%)	9 (30.0%)	5 (20.0%)	27 (14.5%)	9 (90.0%)	8 (100.0%)	2 (40.0%)	8 (38.1%)
**Income group (per person)**								
Low	36 (43.9%)	21 (43.8%)	11 (29.7%)	116 (20.6%)	26 (96.3%)	18 (94.7%)	6 (85.7%)	47 (50.5%)
Middle	18 (32.7%)	16 (34.8%)	4 (36.4%)	52 (14.6%)	14 (93.3%)	12 (85.7%)	2 (66.7%)	21 (48.8%)
High	18 (29.5%)	8 (13.6%)	16 (23.5%)	75 (16.6%)	11 (64.7%)	5 (62.5%)	4 (30.8%)	17 (29.3%)

Those covered by URBMI had less forgone necessary admissions than those without insurance in both years. The proportion of forgone necessary admissions due to financial difficulties was also lower among the URBMI group. Men had a higher reduction in forgone necessary admissions between 2008 and 2013 than women. Age groups 60–69 and 70–79 were more likely to have forgone necessary admissions than those aged 80 and over. The low-income group had the highest number of forgone necessary admissions in 2008 but also had largest decrease in forgone admissions between 2008 and 2013. The proportions of forgone necessary admissions due to financial difficulties decreased among all income groups between 2008 and 2013. Yet the low-income group still had the highest proportion of forgone necessary admissions due to financial difficulties compared to the other income groups (Table 4).

### Factors associated with service utilization

Outpatient visits and hospital admissions significantly increased from 2008 to 2013 (M1–5, Table 5). Compared to the uninsured group, those with URBMI were more likely to have outpatient visits and hospital admissions (OR: 1.23 and 1.57 respectively, M3&4, Table 5). Gender was not found to significantly influence service utilization (M1–5, Table 5). Compared to the group aged 60–69, those aged 70–79 were more likely to have outpatient visits and hospital admissions, while no significant difference was found for those aged 80 and over (M1–5, Table 5). Income level was a significant factor associated with service utilization. The high-income group was more likely to use outpatient and inpatient services than the low-income group (M1–5, Table 5). Meanwhile, the middle-income group only had a significantly higher outpatient services utilization when compared to the low-income group (M1&3, Table 5). Health needs were found to be a determinant for service utilization among the study population who also reported difficulty in taking care of themselves; they were also more likely to use outpatient services and have hospital admissions than those who reported no difficulty with self-care (M1–5, Table 5). Those with higher EQ-5D scores (indicated better health) were less likely to utilize health services. Size of household and marital status did not have a significant impact on service utilization (M1–5, Table 5). Among the insured group, higher education was negatively associated with hospital admission (M2, Table 5) but this association was not significant among the rest of population when we controlled for insurance status (M3&4, Table 5). When controlling for insurance status, those with their own income were less likely to use outpatient services than those relying on other sources of income (M3, Table 5). M5 resulted in similar findings as M4, but when controlling for other factors, individuals with self-reported NCDs were twice more likely to use inpatient services compared to those who did not report NCDs (M5, Table 5).

**Table 5:** Factors associated with health services utilization (OR[95CI])

	M1: Outpatient	M2: Inpatient	M3: Outpatient	M4: Inpatient	M5: Inpatient
	*N* = Insured population		*N* = All population		
Insurance (uninsured as base level)					
URBMI	/	/	1.23 [1.04,1.46]*	1.57 [1.28,1.93]*	1.54 [1.25,1.89]*
Year (2008 as base level)					
2013	1.07 [1.02,1.11]*	1.11 [1.06,1.17]*	1.05 [1.02,1.09]*	1.60 [1.31,1.95]*	1.41 [1.15,1.72]*
Age group (60–69 as base level)					
70–79	1.20 [1.05,1.38]*	1.42 [1.22,1.66]*	1.22 [1.07,1.38]*	1.41 [1.22,1.63]*	1.34 [1.16,1.55]*
80+	0.88 [0.71,1.09]	0.84 [0.66,1.06]	1.00 [0.84,1.21]	0.91 [0.74,1.13]	0.92 [0.74,1.13]
Income level (per person, low as base level)					
Middle	1.25 [1.07,1.47]*	1.14 [0.96,1.36]	1.27 [1.10,1.48]*	1.13 [0.96,1.33]	1.11 [0.94,1.31]
High	1.46 [1.26,1.71]*	1.30 [1.10,1.54]*	1.52 [1.32,1.75]*	1.34 [1.14,1.57]*	1.31 [1.11,1.53]*
Income source (other as base level, such as from children, friends)					
Own	0.87 [0.75,1.00]	1.11 [0.95,1.31]	0.86 [0.75,0.98]*	1.16 [1.00,1.35]	1.15 [0.98,1.34]
Difficulty in self-care in the past month (no difficulty as base level)					
Yes	1.03 [0.83,1.27]	1.59 [1.28,1.97]*	1.19 [1.01,1.41]*	2.15 [1.81,2.54]*	1.98 [1.67,2.35]*
EQ-5D	0.25 [0.17,0.37]*	0.12 [0.08,0.18]*	0.25 [0.17,0.37]*	0.26 [0.21,0.33]*	0.32 [0.26,0.41]*
Self-reported NCDs (no as base level)					
Yes	/	/	/	/	2.54 [2.21,2.91]*
Gender (women as base level)					
Men	0.93 [0.81,1.06]	0.90 [0.78,1.05]	0.95 [0.84,1.07]	0.92 [0.80,1.05]	0.96 [0.83,1.11]
Household size (1-2person as base level)					
3–4	1.00 [0.84,1.18]	0.94 [0.78,1.14]	1.09 [0.93,1.27]	1.05 [0.88,1.25]	1.03 [0.87,1.23]
≥ 5	1.08 [0.91,1.29]	1.07 [0.89,1.30]	1.07 [0.91,1.25]	1.06 [0.89,1.27]	1.08 [0.90,1.29]
Highest education of family (None and primary as base level)					
Secondary	1.09 [0.93,1.28]	0.80 [0.68,0.95]*	1.03 [0.89,1.18]	0.91 [0.78,1.06]	0.91 [0.78,1.07]
College and above	0.98 [0.79,1.22]	0.65 [0.51,0.82]*	1.07 [0.91,1.25]	0.82 [0.65,1.02]	0.82 [0.65,1.02]
Marital status (single or widowed as base level)					
Married	1.05 [0.89,1.24]	0.95 [0.79,1.14]	1.04 [0.84,1.21]	0.93 [0.78,1.10]	0.94 [0.79,1.11]
LR chi2	97.07	324.10	158.77	427.15	615.92
P	< 0.0001	< 0.0001	< 0.0001	< 0.0001	< 0.0001

*p < 0.05

## Discussion

The study found that insurance, self-reported health needs, age and income were all significant factors associated with health service utilization. Health utilization and self-reported health needs of older adults both increased after the implementation of URBMI.

These findings have many policy implications for the delivery and financing of healthcare. Self-reported illness and prevalence of NCDs among older adults increased at an alarming rate between 2008 to 2013. The aging trend and changes of lifestyle of Chinese population have contributed substantially to the increasing prevalence of NCDs. Awareness of NCDs has also been improved significantly by the health promotion and education programs, as well as the health insurance schemes. The implementation of the National Public Health Essential Services since 2009, providing free hypertension and diabetes screening, diagnosis and management to the public at primary health facilities, has proved to increase the timely diagnosis of NCDs as well [22]. Regardless, China’s aging population will require health systems to sufficiently address NCD treatment and prevention. One step would be to ensure public health and primary health providers promote healthy lifestyle education and early screenings for NCDs [23]. Evidence has shown that population-wide policies and interventions such as healthy-lifestyle education is a cost-effective way to prevent and treat NCDs [24]. Primary health providers are an important part of the management and coordination of care for patients with NCDs.

Our finding that health service utilization increased after the implementation of URBMI confirms previous findings from Chen, G. et al. [17]. In addition, forgone necessary admissions and financial difficulties related to forgone necessary admissions decreased, suggesting that URBMI improved access to health services for older adults living in urban China.

However, several challenges remain. First, individuals with URBMI had a higher prevalence of self-reported NCDs than those without insurance, indicating the possible existence of adverse selection in URBMI enrollment. Adverse selection exists widely among voluntary insurance schemes and endangers the financial sustainability of the schemes [25–27]. This is of particular concern with URBMI as its benefits packages and contribution rates vary across provinces [28]. Another interpretation of this finding may be that individuals with insurance have better access to healthcare and therefore are more likely to have had their conditions diagnosed than those without insurance, thus leading to higher self-reporting among the former individuals.

Second, while health insurance improves access to health services, the rationale behind utilizing health services has not been well studied. While we observed that the insured group had a higher hospital admission rate than the uninsured group, there was only a slight difference between these two groups in outpatient visits. In 2008, the insured group had fewer outpatient visits than the uninsured group. This difference could be associated with URBMI’s benefits package which provides a relatively high reimbursement rate for hospital admissions but limited or no reimbursement for outpatient services, this was especially true in 2008 [15]. After controlling for other factors, we found that the insured group were more likely to have outpatient visits and hospital admissions (OR: 1.23 and 1.57, respectively) than the uninsured group. Hospital admissions usually incur higher expenses than outpatient visits and are the major cause of catastrophic health expenditure. Policies determining benefits packages should take this into account and use evidence-based cost-effective treatments and prevention measures, especially with regards to services addressing NCDs. For example, many cities have started to provide higher reimbursement rates for outpatient visits (or more specifically for outpatient visits for certain NCDs). The impact of these policy initiatives should be studied to help inform future policy development and implementation.

Third, despite a decrease in the number of forgone necessary hospital admissions, one-fifth of the study population still had to forgo a necessary hospital admission. In 2013, 40% of these cases were caused by financial barriers. Another national representative survey (CHARLS) for people aged 45 or over found that only 6.8% of the study population reported foregone inpatient care in 2013 [29]. This rate is much lower than our study population, suggesting individuals aged 60 or over have a significantly higher risk of having to forgo necessary hospital admissions than younger populations. Therefore, URBMI should provide better financial protection for older adults. For example, in Hangzhou, older adults receive higher subsidies from the government and have better financial protection through URBMI [7].

Fourth, individuals with higher incomes were more likely to utilize services than those with lower incomes. The middle- and high- income groups with insurance had more outpatient visits and hospital admissions than the low-income group, calling for further attention to be given to issues around equity. Medical Financial Assistance and Insurance Programs for Catastrophic Disease, two programs that targeting at population in poverty and patients with catastrophic health expenditures, should provide more support to low-income groups. Our study clearly demonstrated that there is an increasing demand for health services among older adults living in China’s urban areas. While the implementation of URBMI has helped increase access to services, access has not been equal across all income groups and inequity remains an issue of concern. Prioritizing NCDs and applying cost-effective policies will further improve benefits packages and help to address inequity.

Our study is the first to use the National Household Survey to examine service utilization among older adults in urban areas. While uneven sample sizes in 2008 and 2013, and the fact that the demographic characteristics of the study populations were not homogeneous pose some limitations for the study, they should not bias our major conclusions. A propensity score matching (PSM) approach could identify a matched sub-sample from the study population and balance the sample sizes between different groups [30], however, this would further reduce the relatively small sample size for 2008 survey and there are limited choices for variables to be used for matching. Additionally, considering the implementation process of URBMI, the enrollment decision on URBMI was different between 2008 and 2013 sample. For study population from 2008 survey, URBMI was just implemented for few months, while for study population in 2013, URBMI has scaled up to all urban areas in China and the total population coverage rate was over 90%. We have reason to believe that the health needs and services use of study populations from 2008 and 2013, were jointed influenced by individual decision and the policy background. After matching, we would not be able to present such impact and therefore, we decided to report findings from the original study population without applying PSM.

## Conclusion

Health needs among older adults increased alarmingly between 2008 and 2012, especially those related to NCDs, implying that NCDs should be made a high priority. Health services utilization increased and forgone necessary admissions decreased after the implementation of URBMI, indicating an improvement in access to health services for the study population. However, higher-income groups appeared to have better access than lower-income groups. To help address issues around inequity, insurance benefits packages should provide more support for the prevention and treatment of NCDs.

## Data Availability

The original data from the National Health Services Surveys is not available due to National Health Commission regulations. For questions or inquiries, please contact Centre for Health Statistics and Information of the National Health Commission.

## Abbreviations

CPI: Consumer Price Index
EQ-5D: EuroQol five dimensions questionnaire
OR: Odds ratio
NCD: Non-communicable disease
NCMS: New Cooperative Medical Scheme
NHSS: National Health Services Survey
UEBMI: Urban Employees’ Basic Medical Insurance
UHC: Universal health coverage
URBMI: Urban Residents’ Basic Medical Insurance
